# TONSILECTOMIES DRIVE

**DOI:** 10.1016/S1808-8694(15)30094-X

**Published:** 2015-10-19

**Authors:** Ivan D. Miziara

**Affiliations:** Associate Professor – University of São Paulo Medical School Physician in charge of the ENT Clinics University of São Paulo Medical School – HC-FMUSP.

Since the first tonsillectomy performed in São Paulo by Schmidt Sarmento, in 1920 at the Santa Casa de São Paulo Hospital, this type of surgery has been used to solve the problem of chronic infections and obstruction caused by the palatine tonsils, and it is becoming one of the most common surgical procedures performed in the world. In the United States alone, it is estimated that about 250 thousand of such procedures are performed annually to remove tonsils and adenoids.

Things are not different in Brazil. Although we do not have solid figures, hundreds of thousands of children and adults are submitted (or should be) to tonsillectomies. For this reason, and the very difficulties inherent to our public health care system, the lines of patients awaiting surgery in public and /or university hospitals are huge. In many a case, people have to wait up to two years for the procedure.

In an attempt to solve this problem of long waiting lines, when José Serra headed the Ministry of Health, many surgical procedures were performed in public hospitals in the mode of a joint effort. In the following years, such events became routine, and are performed in different sites of the country. In our University Hospital at the University of São Paulo Medical School, we performed two of these tonsillectomy drives, in 2005 and 2006.

In total, 400 patients (mostly children) were operated along these two years. All of them by the dissection approach under general anesthesia with oro-tracheal intubation. Incidents such as post-operative bleeding were not higher than what is described in the medical literature (about 5% in average). Most of the patients were discharged on the same day, without major problems.

After a careful assessment of the tonsillectomies drive carried out in our institution - HC-FMUSP, the result was very positive. The waiting line was reduced to practically two months. Surgical teaching was enhanced and the participation of the assistant-physicians during all the process, working as volunteers, was above our expectations.

Unfortunately, for the year of 2007, we have not scheduled a joint effort in tonsillectomies in our University Hospital. There are a number of reasons for it, most of them related to the State and Municipal Secretaries of Health, disregarding the willingness of our ENT Department, headed by Professor Ricardo Ferreira Bento, a strong advocate of these surgical marathons.

Results: today we have over 400 patients (adults and children) waiting in line for the procedure.

As we see it, surgical drives like this one are fundamental within the current public health care framework of our country. Especially considering university hospitals, which receive patients from all corners of our country, without following rules of referral by region.

The waiting line, in itself, is something inhuman and a form of violence against the needy population, who depend on public hospitals to solve their health problems.

Evidently, these drive are not the ideal solution, but one that is possible. And between doing nothing and doing what is possible, logic and common sense tells us to chose the latter. In order to be carried out with some frequency, we only need the support from the health secretaries, because physicians are willing to do their part.

Now, I yield the floor to our health care administrators.

